# Therapeutic angiogenesis-based strategy for peripheral artery disease

**DOI:** 10.7150/thno.74785

**Published:** 2022-06-27

**Authors:** Jingxuan Han, Lailiu Luo, Olivia Marcelina, Vivi Kasim, Shourong Wu

**Affiliations:** 1 The Key Laboratory of Biorheological Science and Technology, Ministry of Education, College of Bioengineering, Chongqing University, Chongqing 400044, China.; 2 State and Local Joint Engineering Laboratory for Vascular Implants, Chongqing 400044, China.; 3 The 111 Project Laboratory of Biomechanics and Tissue Repair, College of Bioengineering, Chongqing University, Chongqing 400044, China.

**Keywords:** therapeutic angiogenesis, peripheral artery disease (PAD), critical limb ischemia (CLI), vascular regeneration, angiogenesis factors

## Abstract

Peripheral artery disease (PAD) poses a great challenge to society, with a growing prevalence in the upcoming years. Patients in the severe stages of PAD are prone to amputation and death, leading to poor quality of life and a great socioeconomic burden. Furthermore, PAD is one of the major complications of diabetic patients, who have higher risk to develop critical limb ischemia, the most severe manifestation of PAD, and thus have a poor prognosis. Hence, there is an urgent need to develop an effective therapeutic strategy to treat this disease. Therapeutic angiogenesis has raised concerns for more than two decades as a potential strategy for treating PAD, especially in patients without option for surgery-based therapies. Since the discovery of gene-based therapy for therapeutic angiogenesis, several approaches have been developed, including cell-, protein-, and small molecule drug-based therapeutic strategies, some of which have progressed into the clinical trial phase. Despite its promising potential, efforts are still needed to improve the efficacy of this strategy, reduce its cost, and promote its worldwide application. In this review, we highlight the current progress of therapeutic angiogenesis and the issues that need to be overcome prior to its clinical application.

## Introduction

Peripheral artery disease (PAD) has always been a global health issue affecting people's lives and quality of life 1. PAD is indicated by the presence of stenosis or occlusion in the peripheral artery, mainly in the lower extremities 2. In 2015, more than 235 million people aged 25 years or older were estimated to suffer from PAD 3. The prevalence of PAD increases along with age and the presence of several important risk factors, including smoking, diabetes, hypertension, and dyslipidemia. As the number of projected individuals with the aforementioned risk factors increases, the number of PAD patients is expected to rise in the coming years 1.

Critical limb ischemia (CLI) is the most severe clinical manifestation of PAD. Limb ischemia stands for the pathological changes in blood vessels in patients' feet, resulting in insufficient blood supply, especially in the lower extremity 4. In severe cases, CLI might lead to tissue necrosis, amputation, and even mortality 5-7. Previous clinical studies have revealed that 20% of patients with chronic CLI would die within the first year of symptom onset, while 22% of them would experience major amputation 5, 8. Mortality rates increased from 50% to 65% within five years, exceeding the five-year mortality rate of other diseases such as myeloma and colon cancer 9, 10. Furthermore, patients with PAD have an increased risk of coronary and cerebrovascular disease-related morbidity and mortality 11. However, despite its high prevalence and serious implications, PAD remains relatively underdiagnosed 3, 12, 13.

Surgical-based revascularizations that aim to provide a blood flow in the ischemic tissue through direct interventions in the affected vasculature, such as implantation of the catheter, vascular stent, or balloon, are currently the first choice and the standard strategy for restoring blood perfusion in PAD patients 14, 15. While these strategies could reach 60% to 80% efficacy in correctly selected patients 16, there are several limitations to their application: (1) the optimal timing and indications remain controversial; (2) some patients may suffer from severe postoperative complications; and (3) some patients are not suitable for revascularization 16-19. Indeed, 20 to 40% of patients with CLI could not receive surgical-based revascularization treatments due to already severely damaged vessels, and many of these “no-option” patients required amputation to prevent the generation of other complications 19, 20. Furthermore, even after revascularization, 10% of patients are expected to be hospitalized again due to major adverse limb events (MALE) within one year 21, leading to a poor prognosis 22. Amputation of the lower extremities is not only associated with lower survival time, but it also has a significant impact on patients' quality of life, disturbing their mobility and daily activities 23.

Therapeutic angiogenesis, which aims to induce endogenous vessel formation and blood perfusion recovery in the ischemic sites, is considered one of the most promising methods for treating no-option PAD patients 24, 25. Utilizations of gene-, cell-, protein-based therapy, small molecule drugs, and combinatorial therapies have been studied for more than two decades; however, due to the lack of benefit and efficiency in clinical trial results, these approaches have not been established as a widely used strategy for PAD patients 24, 26. Currently, there are only two commercially available therapeutic strategies for PAD, both of which are gene-based. One of them uses non-viral supercoiled plasmid encoding vascular endothelial growth factor (VEGF), which was approved in Russia in 2011 and then in Ukraine in 2013 27, 28. In contrast, the other uses a viral vector encoding hepatocyte growth factor (HGF), which was recently approved in Japan in 2019 29. However, their expensive costs hinder their use worldwide. Hence, low-cost, effective agents for inducing effective therapeutic angiogenesis are urgently needed. In this review, we will discuss the molecular mechanism underlying PAD and the current progress in therapeutic angiogenesis using different approaches.

## Peripheral artery disease

In the human vascular system, a single blood flow of the aorta is bifurcated into two main arteries to further provide the bloodstream to each leg. From this point, the artery from each leg is branched into several arteries in the tibial area, with one main artery serving blood flow to the distal leg. Peripheral artery disease is a pathological condition caused by an obstruction in blood flow, mainly caused by the formation of plague and/or damage to blood vessels. Formation of plaque, for example, due to the accumulation of low-density lipoprotein or dead cells inside blood vessels, could lead to platelets and eventually atherosclerosis, resulting in vessel restriction or occlusion 30. Dysfunction or even cell death of blood vessel-forming cells, such as endothelial cells (ECs) and vascular smooth muscle cells (VSMCs) is also a significant cause of PAD. This could be caused by the increase of reactive oxygen species (ROS) and advanced glycation end-products (AGEs) as a result of risk factors such as smoking, diabetes, hypertension, and aging. Occlusion in the vessel disrupts the blood flow and interrupts the deliveries of oxygen and nutrients inside the blood vessel, further impairing ECs and VSMCs survival and functions by causing oxidative stress 31-34. Besides ECs and VSMCs, PAD is also associated with the damage of other cells involved in angiogenesis 35. Macrophage, especially its anti-inflammatory state (M2 phenotype), is crucial for angiogenesis, as it could induce wound healing by secreting profibrotic factors that contribute to tissue repair. However, damage-associated molecular signals released during the progression of PAD induce macrophages polarization into the pro-inflammatory state (M1 phenotype), further contributing to cellular damages 36. PAD could also lead to fibrosis in red blood cells, the most abundant cells in the circulating blood, causing them to collide with the arterial wall, inducing local retention of lipid from the red blood cells membrane and local hemolysis. Ruptured red blood cells could also release heme-ferrous ions with high toxicity for ECs and VSMCs, further contributing to ECs and VSMCs dysfunction and death 37, 38. Furthermore, the accumulation of these dead cells could also induce plaque formation, thus further contributing to advanced PAD. As the total occlusion of this single continuous vessel will lead to PAD, the blood supply will be dependent on collateral vessels 24.

PAD is classified as asymptomatic or symptomatic, and both can be diagnosed using lower ankle-brachial index (ABI) measurement as an indicator 12, 39. However, since patients with asymptomatic PAD have not yet experienced symptoms such as pain or claudication, the lack of noticeable symptoms hinders early intervention of PAD clinically. Furthermore, the relationship between ABI screening and improved health outcomes in asymptomatic adults is limited 39, 40, putting patients with asymptomatic PAD at risk of morbidity and mortality 41-43. Although asymptomatic PAD is mostly unnoticed and draws less attention in most studies, its occurrence contributes to the most among PAD prevalence. Moreover, it is associated with a significantly poorer prognosis than vascular diseases in other territories 12, 44. As the risk of disease progression from the milder manifestation of PAD to the more severe state has been underestimated in previous studies 45, new tools or investigations should be adopted to screen for asymptomatic PAD 39-41, 43.

In symptomatic PAD, the degree of manifestation ranges from intermittent claudication (IC) to CLI. IC, which is indicated by exercise-induced limb discomforts, such as pain, fatigue, and cramping, is primarily a quality-of-life disease that could be relieved by resting. Meanwhile, people with CLI suffer from limb discomfort, even at rest, with or without the presence of ulcers or gangrene 2, 6. While amputation occurs in 4 to 27% of IC patients 45, 30% of CLI patients will undergo alive amputation within one year. Moreover, 50% of CLI patients will eventually die within five years of their diagnosis, increasing the burden of CLI treatment 8, 20.

PAD is also one of the major complications in diabetes, as hyperglycemia could induce systematic impairment in cellular functions, including paracrine function, proliferation, and migration potential 46, 47. Hyperglycemia-induced AGEs impair cell functions and, subsequently, angiogenic potentials in diabetic PAD patients, leading to severe outcomes and a poor prognosis, with a wider damaged area and a higher reoccurrence rate 48. Hyperglycemia also causes severe damage to skeletal muscle tissues, which are the largest secretory organ in the human body that could secrete a myriad of factors, including cytokines and angiogenic growth factors, involved in the neovascularization process 49-51. Indeed, a previous study has revealed a correlation between the poor prognosis of limb ischemia disease with the loss of skeletal muscle capillary density 52. Furthermore, AGEs reduce mitochondrial efficiency in myoblasts and the expression of myogenic regulatory factors, thus increasing skeletal muscle cell death while reducing muscular strength in diabetic patients 53-55. These factors, taken together, constitute the main obstacles to inducing effective therapeutic angiogenesis in diabetic PAD patients 49, 56, 57.

## Molecular mechanism of vascular regeneration in PAD

**Figure 1:**
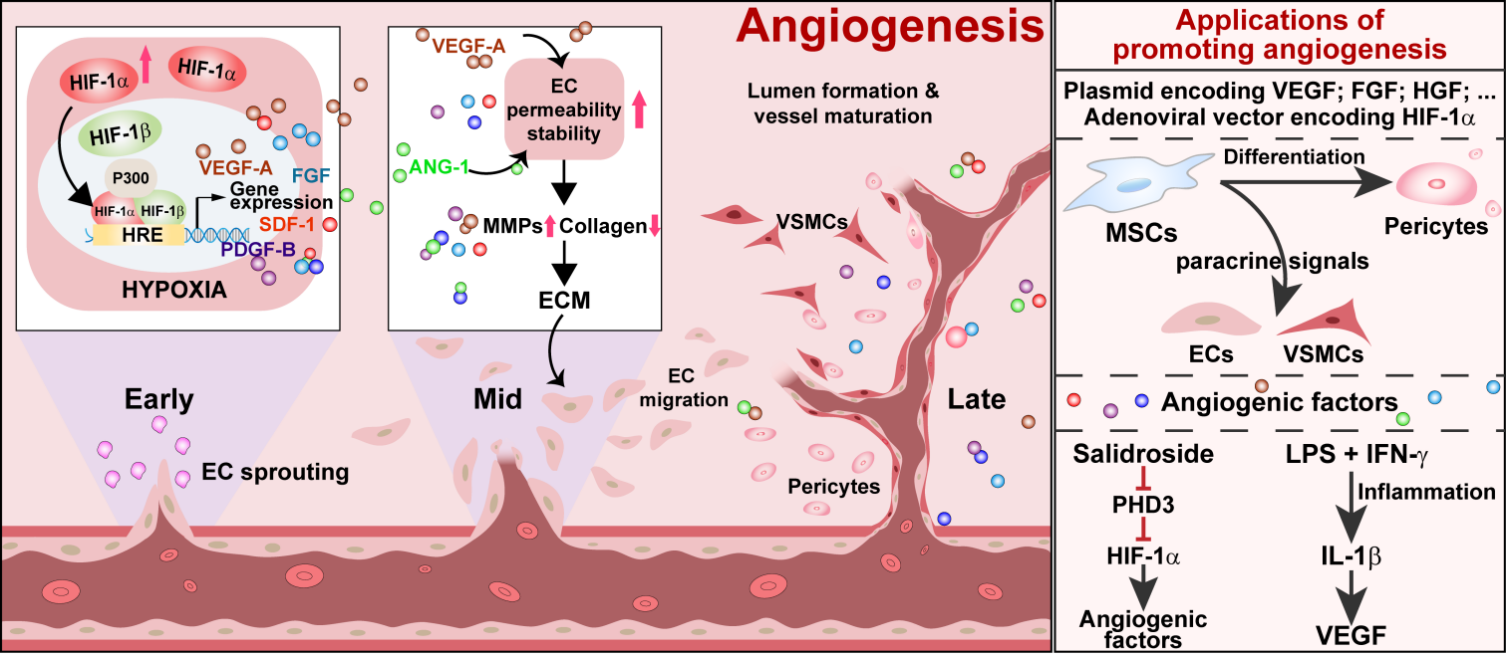
** Schematic diagram of ischemic/hypoxic-triggered angiogenesis and its applications.** Accumulated HIF-1α triggers the expression of various angiogenic factors by binding with the HIF-1 response element (HRE) on their promoters. These angiogenic factors promote endothelial cells permeability as well as stability, thus enhancing the process of endothelial sprouting from the existing vessel. Vascular smooth muscle cells and pericytes assist in new blood vessel formation and maturation to promote angiogenesis. ECs: endothelial cells; VSMCs: vascular smooth muscle cells.

Angiogenesis is the formation of new blood vessels from an existing vascular structure. It is a complex process involving multiple angiogenic factors and various types of cells (**Figure 1**). In the early stage of angiogenesis, the expression of VEGF-A, fibroblast growth factor-2 (FGF-2), and HGF are stimulated. VEGF-A promotes ECs permeability. At the same time, it stimulates pericytes to express matrix metalloproteinases (MMPs), which degrades collagen, vascular basement membrane, and extracellular matrix (ECM). FGF-2 stimulates ECs to produce both MMPs and VEGF-A and ECs proliferation, pericyte attraction, and ECM deposition in the ischemic site 58. HGF further stimulates VEGF-A expression, ECs growth, and proliferation 59, 60. These events lead to the extravasation of plasma proteins from ECs to generate a provisional ECM, which, in turn, supports the migration of ECs to sprout from the existing vessel and form a vessel lumen 61, 62. However, up to this stage, vessels are formed merely by ECs and are immature and leaky. Coverage by VSMCs and pericytes is needed for the maturation and stabilization of vessels. Angiopoietin-1 (ANG-1) produced by mural cells assists in this process, as it could activate its endothelial-specific tyrosine kinase receptor Tie2. Activated Tie2 promotes vessel assembly and maturation by mediating survival signals for ECs and regulating the recruitment of mural cells 63, thus stabilizing blood vessels and promoting their leak resistance by tightening endothelial junctions 64. Platelet-derived growth factor-BB (PDGF-BB) stimulates the stabilization of newly formed vessels by inducing the alteration, proliferation, migration, and recruitment of pericytes and VSMCs associated with ECs-induced sprouts 62, 65, 66. Subsequently, these angiogenic factors will trigger the proliferation and migration of mural cells, consisting of VSMCs and pericytes 67. These cells are recruited to cover the EC-formed vessel lumen, leading to the formation and stabilization of a functional blood vessel.

**Figure 2:**
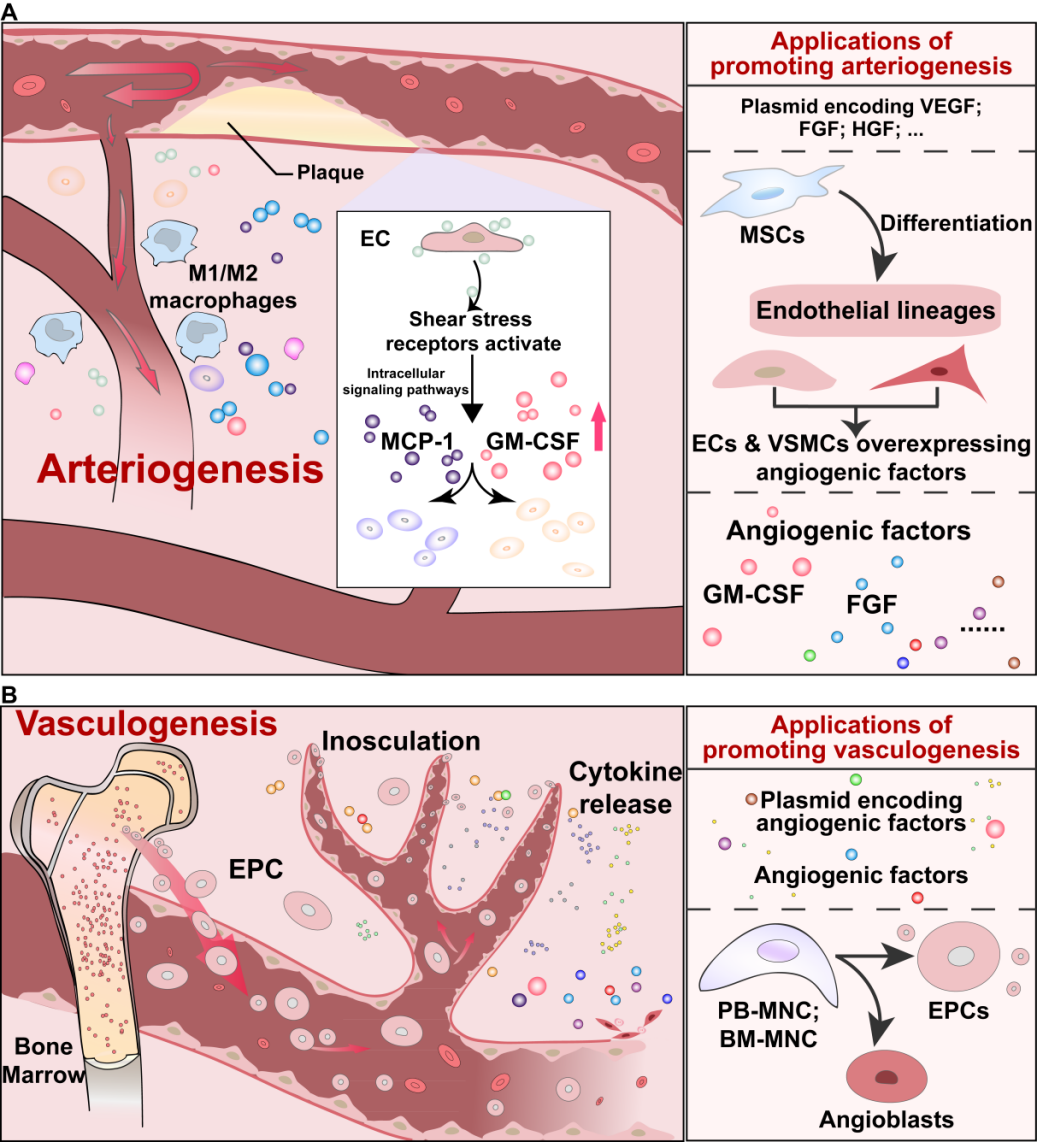
** Schematic diagrams of arteriogenesis and vasculogenesis, and their applications. (A)** Signaling pathways of arteriogenesis that contribute to vessel remodeling, and applications to induce arteriogenesis. **(B)** Signaling pathways of endothelial precursor cells-mediated vasculogenesis, and applications to promote vasculogenesis. EPC: endothelial precursor cell.

The arteriogenesis process will also be induced in response to arterial blockage irrespective of the ischemic condition. As shown in **Figure 2A**, in the arteriogenesis process, increased shear stress and inflammation due to arterial occlusion leads to the formation of collateral vessels. Shear stress receptors on ECs activate the downstream intracellular signaling pathways and release several important cytokines, such as monocyte chemoattractant protein 1 (MCP-1), granulocyte-macrophage colony-stimulating factor (GM-CSF), and cell adhesion molecules. Subsequently, monocytes, which produce proteases, and other white blood cells, which produce growth factors, such as FGFs, will be recruited to mediate vessel remodeling 24, 68.

In addition to angiogenesis and arteriogenesis, vasculogenesis, which is activated by endothelial progenitor cells (EPCs), is another process of neovascularization (**Figure 2B**) 69, 70. Vasculogenesis was previously known to occur only during the embryonic developmental stage, as EPCs could physically adhere to the endothelium, integrate into blood vessels, and support angiogenesis via their paracrine actions. However, Asahara *et al.* and Shi *et al.* showed the presence of EPCs in adult peripheral blood and bone marrow, respectively 71, 72. Mainly circulated from bone marrow, EPCs could reach the affected vessels and stimulate neovascularization through paracrine function 24.

While the interplay of angiogenesis, arteriogenesis, and vasculogenesis is important for restoring limb function, these processes are disrupted in PAD patients due to microvascular and endothelial dysfunction 73. Generally, obstructive lesion in the lower limb artery is generated by atherosclerosis and it is worsened by the concurring state of the patient itself, such as hyperglycemia, hypertension, and dyslipidemia.

PAD also leads to the ischemic condition, causing systemic damage to blood vessels as well as other cells that support angiogenesis, for example, skeletal muscle cells, which secrete various angiogenic factors, such as ANG-1, VEGF-A, and PDGF-BB, all of which are crucial in inducing angiogenesis 49, 51. However, while the damage caused by PAD is usually severe, previous studies using hindlimb ischemia (HLI) model mice have shown that blood perfusion in mild HLI could recover 56, 74, 75, indicating the presence of revascularization and self-recovery innate potential. Indeed, hypoxic condition triggers the activation of hypoxia-inducible factor-1 (HIF-1) signaling, which is responsible for the cellular response to acute hypoxic stress 76, 77. Under the normoxic condition, HIF-1α, one of the two subunits forming HIF-1, is hydroxylated by the prolyl-hydroxylase domain (PHD) family, especially PHD-2 and PHD-3, using oxygen as its substrate 78, 79. This hydroxylation leads to ubiquitination and, subsequently, the proteasomal degradation of the HIF-1α protein 80, 81. When cells are exposed to hypoxic conditions, the PHD family fails to hydroxylate HIF-1α due to the lack of oxygen, resulting in its accumulation 82, 83. This, in turn, enhances the HIF-1α heterodimer formation with another HIF-1 subunit, HIF-1β, and subsequently stimulates the transcription of HIF-1 target genes. More than 160 genes have been identified as HIF-1 targets 84, 85, including those involved in angiogenesis such as VEGF-A, PDGF-BB, stromal cell-derived factor-1 (SDF-1), and endothelial nitric oxide synthase (eNOS) 86-89, as well as those involved in cell survival such as heme oxygenase-1 (HO-1) 90, 91. However, this innate potential is frequently insufficient to cover the damage induced by PAD, especially in patients with severe blood vessel damage, such as those with CLI or diabetes 24, 25, 57, 92.

Hence, promoting innate angiogenic potential, which leads to increased expression of angiogenic factors, has emerged as an attractive strategy for enhancing the neovascularization process in PAD patients. Evidently, as discussed below, attempts using different approaches have been made to establish potential strategies for treating PAD by promoting patients' innate angiogenic potential.

## Trends in therapeutic angiogenesis for PAD

**Figure 3:**
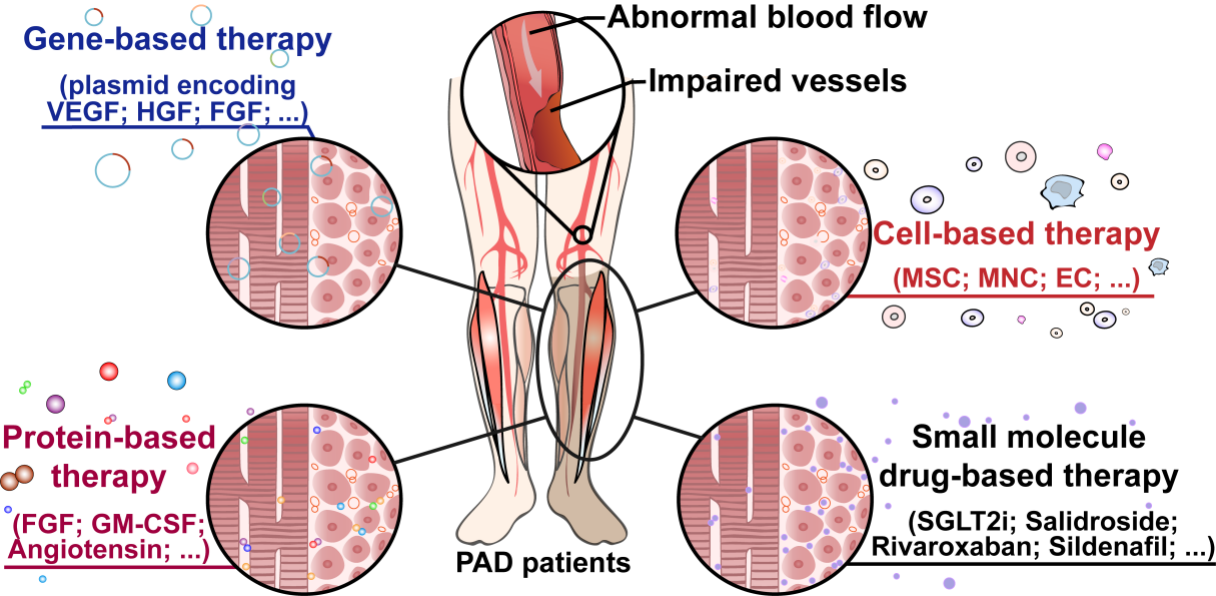
** Therapeutic angiogenesis-based strategy for peripheral artery disease.** Schematic diagram of regenerative approaches for treating peripheral artery disease and their applications. PAD: peripheral artery disease.

For approximately two decades, “therapeutic angiogenesis” has been studied as an investigational approach to treat symptomatic PAD, especially for “no-option” patients 24-26. Therapeutic angiogenesis aims to stimulate vessel formation and blood perfusion to alleviate hypoxic damages caused by insufficient oxygen supply into organs and tissues due to ischemia 24. Since the concept of therapeutic angiogenesis first appeared in 1994, multiple approaches to ischemic diseases have been investigated 61, 93. The first attempt at therapeutic angiogenesis was performed by Takeshita *et al.* in 1994. They intraarterially administered VEGF in HLI rabbits and observed the development of angiographically visible collateral arteries 61. The first human trial of therapeutic angiogenesis for limb ischemia was conducted by Isner *et al.* in 1996. They used a hydrogel-coated balloon-angioplasty-catheter to intraarterially transfer a plasmid encoding a soluble 165-amino acids isoform of human VEGF-A protein (*VEGF-A_165_*) into a patient with an ischemic right leg and observed an increase in collateral vessels and blood flow, suggesting that the treatment promoted angiogenesis in the ischemic limb 94. Following this clinical study, exploration studies into therapeutic angiogenesis have been carried out through different approaches, including gene-, cell-, and protein-based therapy, as well as small molecule compound therapy (**Figure 3**).

### Gene-based therapy

Gene-based therapy is a method of increasing revascularization by injecting genetic materials encoding target genes into ischemic sites in patients. Generally, the introduction of genetic material into a host can be achieved by using non-viral vectors, such as plasmid DNA or viral vectors, such as adenovirus and Sendai virus 95, 96. Since the first clinical trial for VEGF was conducted in 1996, extensive studies have been performed to assess the effectiveness of other pro-angiogenic factors, such as HGF and FGF (**Table 1**) 29, 97-101. Furthermore, many studies using other pro-angiogenic factors, such as angiogenic factor with G-patch and Forkhead-associated domain 1 (AGGF1), human telomerase reverse transcriptase (hTERT), and some microRNAs (miRNAs), also showed promising results in promoting angiogenesis in animal models 102-105.

VEGF-A is one of the most studied factors in angiogenesis-related diseases due to its critical role in initiating the angiogenesis process 68. A phase I clinical trial established by Rajagopalan *et al.* demonstrated that intramuscular injection of an adenoviral vector encoding the 121-amino acid isoform of *VEGF-A* into PAD patients with IC or rest pain (RP) may favorably influence lower-extremity endothelial function 106, 107. A drug containing *VEGF-A_165_*-expressing plasmid called Neovasculgen was approved in Russia in 2011 and Ukraine in 2013. Neovasculgen targets a spectrum of patients with mild to severe claudication due to CLI, yielding a significant increase in pain-free walking distance in a five-year follow-up study and five-year post-marketing surveillance 27, 28. While Deev *et al.* claimed that no gene transfer-related adverse events or side effects were observed, other information about this drug is limited, as it is currently only approved in Russia and Ukraine 27, 28. However, a clinical trial established by Isner *et al.* found that angiogenesis in patients' ischemic sites promoted by VEGF-A failed to prevent the need for limb amputation five months after the gene transfer, probably because the blood vessel lumen induced by VEGF-A is immature and leaky 62, 94. Attention should also be paid to the adverse events of this strategy, as the development of spider angiomata and edema was observed in the patient's limb distal to the site of *VEGF-A* gene transfer, possibly due to the effect of VEGF on vascular permeability 106, 108, 109.

HGF, another well-known potent mitogen for endothelial cells, is also an attractive target for inducing angiogenesis. In a phase III clinical trial conducted in 2010, intramuscular-injected plasmid DNA expressing human *HGF* maintained limb perfusion, promoted complete ulcer healing rate and decreased RP in patients with CLI 98, 99. Furthermore, clinical trials conducted in Japan also demonstrated that intramuscular injection of plasmid encoding *HGF* tended to improve RP and decreased the size of the ischemic ulcer in patients with CLI 29, 110, 111, and this gene-based therapy has attained marketing approval for CLI patients in Japan in March 2019. In China, another clinical trial for CLI was conducted using an intramuscular injection of plasmid encoding human *HGF*, pUDK-HGF. The results of its phase II trial in 2014 showed complete pain relief or pain reduction in patients with CLI 97, prompting the recruitment of phase III clinical trial participants (China Clinical Trial Registry, unique identifier: CTR20181274). Several clinical trials involving the combination of human *HGF* isoforms are also being held. For the treatment of PAD using intramuscularly-injected VM202, a plasmid encoding two human HGF isoforms *HGF_723_* and *HGF_728_*, phase II clinical trial recruitment is currently underway (NCT03363165). Meanwhile, Pyun *et al.* conducted a preclinical study in rabbit HLI models using a plasmid encoding the 728 and 723 amino acids isoforms of *HGF*, namely *HGF_728_* and *HGF_723_*, respectively, and discovered that intramuscular injection of *HGF* efficiently increased the number of angiographically recognizable collateral vessels and blood flow 112. Currently, NL003, a plasmid containing novel genomic cDNA hybrid human *HGF* encoding *HGF_728_* and *HGF_723_*, has already passed the phase II clinical trial and is in the process of recruiting CLI patients for the phase III clinical trial (NCT04274049).

Other clinical trials based on gene therapy using angiogenic factors have been conducted using plasmids encoding *FGF*s 113. Nikol *et al.* found that intramuscular injection of plasmid encoding human *FGF-1,* namely NV1FGF, into CLI patients reduced the risk of all amputations, major amputations, and death 113. SeV-hFGF2/dF, a recombinant Sendai virus encoding the human *FGF-2* gene, has currently advanced into phase I clinical trials and is recruiting patients with peripheral arterial occlusive disease (NCT03668353).

While still in preclinical studies, AGGF1 has gained attention as a new angiogenic factor that could effectively promote angiogenesis in HLI animal models 102, 103. Lu *et al.* found that intramuscular injection of plasmid encoding human AGGF1 increased the blood flow and the density of ECs-induced vessels in the ischemic hindlimb of HLI mice 114. Yao *et al.* revealed that AGGF1 activated the antioxidative processes in EPCs to inhibit reactive oxygen species generation, thus promoting EPCs function in diabetic HLI model mice 115. Moreover, Wang *et al.* reported integrin α5β1 as a receptor for AGGF1 in ECs could promote ECs adhesion, migration, capillary tube formation, and therapeutic angiogenesis in the HLI mouse model 116.

While the abovementioned studies focus on angiogenic factors, hTERT is also being assessed in clinical trials for its efficacy in PAD. hTERT is an important component of telomere elongation and thus is expected to ameliorate telomere dysfunction in aging patients in whom PAD mostly occurs. Previous studies have shown that hTERT is one of the VEGF-A downstream effectors and is essential in mediating the angiogenic process *in vivo*. Early preclinical studies demonstrated that overexpression of *hTERT* could enhance the regenerative properties of EPCs 117 and capillaries formation in the HLI rat model 104. Recruitment of CLI patients for a phase I clinical trial using an adeno-associated virus expressing *hTERT* was conducted in 2019 (NCT04110964).

Several gene-based therapies have emerged into clinical trials or even have been approved; however, many of them did not significantly decrease mortality or amputation rates in patients with PAD 118. Along with the insufficient efficacy of gene transfer, the complexity of the neovascularization process, which involves various angiogenic factors and cell types, is also considered one of the main underlying reasons for this failure. For this reason, inducing the expression of multiple angiogenic factors simultaneously has become an attractive strategy. This leads to the development of a second-generation therapeutic angiogenesis strategy, in which multiple angiogenic factors are combined. In a phase I trial for diabetic CLI patients, Barc *et al.* used a plasmid encoding *VEGF-A_165_/HGF* genes, and the results showed that intramuscular injection of this plasmid significantly decreased patients' RP and improved vascularization 119.

Apart from the overexpression of certain genes, the regulation of certain miRNAs has emerged as another potential approach. Numerous miRNAs have been linked to PAD development, suggesting their potential roles for PAD diagnostic and therapeutic targets 120. A preclinical study showed that inhibiting miR-15a/16 with an adenovirus-based decoy system improved angiogenesis in the HLI mouse model through tyrosine kinase receptor upregulation 105. Another preclinical study reported that injecting let-7g intramuscularly caused neovascularization and blood perfusion in HLI mice 121. Meanwhile, overexpression of miR-210 enhanced angiogenic ability in the HLI mice model 122. Another interesting gene delivery approach using mRNA was done by Gan *et al.* Although the phase I clinical trial was not specifically conducted in PAD-related patients, intradermal administration of modified mRNA encoding *VEGF-A_165_* in men with type 2 diabetes mellitus could induce VEGF-A protein levels and skin blood flow 123.

Given that HIF-1α could regulate the transcription of multiple angiogenic factors while enhancing cell survival under hypoxia, attempts have been made to stabilize its protein and increase its accumulation. We previously designed shRNA expression vectors targeting murine PHD2 (shPHD2) and found that subcutaneously-injected matrigel-encapsulated shPHD2 plasmid induced the expressions of multiple angiogenic growth factors by stabilizing HIF-1α protein in mice models 124. We also discovered that hydrodynamic limb vein injection of naked shPHD2 plasmid significantly promoted the formation of mature and functional blood vessels in the ischemic site of the HLI mice model, resulting in enhanced blood perfusion recovery 125. Intramuscular injection of adenoviral vector encoding HIF-1α (Ad2/HIF-1α/VP16) efficiently increased collateral blood vessel formation and blood flow in rabbit HLI and mice diabetic HLI models 126, 127. In a phase I clinical trial, intramuscular injection of Ad2/HIF-1α/VP16 reduced RP and promoted ulcer healing in some patients 89; however, in a phase III clinical trial, an increase in edema rate was observed, probably due to continuously activated HIF-1α 89. Furthermore, no differences in ABI and quality-of-life measurements were found in IC patients who received Ad2/HIF-1α/VP16 injections. Several reasons have been proposed for these unsatisfactory results, including low expression levels of the coxsackie-adenovirus receptor in skeletal muscle in patients 95. The age of patients recruited in the phase III trial, which ranged from 40 to 80 years old, might also affect the outcome of the clinical trial, as a previous study revealed that aging is associated with reduced HIF-1α activity and decreased expression of angiogenic growth factors, as well as attenuated angiogenesis 128. Moreover, the selection of model animals is also assumed to be a critical factor underlying the failures of not only HIF-1α clinical study but also other early therapeutic angiogenesis studies. In early preclinical therapeutic angiogenesis studies, New Zealand white rabbit and C57BL/6 mice, which possess strong self-recovery abilities, were used as the HLI animal models to examine the efficacy of Ad2/HIF-1α/VP16 in inducing therapeutic angiogenesis, leading to a gap between animal and clinical studies 89, 95. Thus, to narrow the gap between preclinical and clinical studies, selecting appropriate model animals, for example, Balb/c mice with poor angiogenic potential, is necessary. Moreover, the fact that Ad2/HIF-1α/VP16 demonstrated positive effects only in part of CLI of patients in phase I clinical trial suggested that the genetic background of the patients, as well as the stage of disease progression, are also critical in the clinical outcome of this approach 89. Thus, while further preclinical and clinical studies are still necessary, the determination of specific criteria of the patient's genetic background and the disease progression might be crucial for determining the appropriate therapeutic angiogenesis strategy for each patient.

Together, gene-based therapies using single factor or multi-factors demonstrate positive effects in promoting therapeutic angiogenesis in preclinical experiments and early stages of clinical trials. However, most gene-based therapies have not progressed into larger-scale clinical trials, and for those that have entered phase II or III clinical trials, many of the results did not meet the previous expectations, suggesting that improvement of delivery efficacy, as well as criteria for selecting appropriate patients according to their genetic background and disease progression, are needed. Meanwhile, although two drugs based on gene therapy have already been approved to treat PAD clinically, their effect on severe CLI remains to be investigated in a larger clinical trial or post-marketing surveillance. Furthermore, the socioeconomic burdens due to their high costs might hinder their global application. Thus, efforts should also be made to reduce the cost of this approach.

### Cell-based therapy

Cell-based therapy requires the administration of viable stem or progenitor cells into a host to induce angiogenesis, either directly through their roles in vessel formation or through the paracrine signaling exerted by the respective cells 129. Since the first clinical trial of this therapeutic strategy using autologous bone marrow-derived mononuclear cell (BM-MNC) in 2002 130, several studies have been conducted in preclinical and clinical settings using various types of cells, such as mesenchymal stem cells (MSCs), MNCs, marker-specific cells, and bone marrow aspirate, to treat PAD 131, and some of them have emerged into clinical trials (**Table 2**). Cell-based therapy could be classified into two types based on the source of the cells used: allogeneic, which uses cells from the healthy donor, and autologous, which uses cells from the corresponding patient directly. The advantages and disadvantages of allogeneic and autologous cell therapies are shown in **Table 3** 132, 133.

Stem cell transplantation has been recognized as a potential strategy for inducing angiogenesis 134, 135. MSCs have proliferation capabilities and differentiate into endothelial lineages. They also contribute to tissue repair and regeneration in acute and chronic skeletal muscle damage by migrating to ischemic tissues. Furthermore, they could secrete paracrine signals that affect other cells, such as ECs and VSMCs, and subsequently promote blood vessel formation and induce therapeutic angiogenesis in ischemic tissues in HLI animal models 136. Several therapeutic angiogenesis efforts using MSCs are currently in clinical trial stages. Gupta *et al*. conducted a phase II clinical trial in which allogeneic MSCs were intramuscularly injected into the gastrocnemius muscle of CLI patients. The results showed that patients who received a large amount of allogeneic MSCs (2 million cells/kg) experienced ulcer healing and a significant reduction in RP 137. Meanwhile, mesenchymal-like cells derived from the allogeneic placenta have also been tested in early clinical trials and showed promising results, such as reduced pain and increased tissue perfusion, leading its way to phase III clinical trial 138.

MNCs derived from adult peripheral blood (PB-MNCs), or bone marrow, are another promising candidate for inducing therapeutic angiogenesis, as they comprise a subset of EPCs and angioblasts, which are beneficial for vasculogenesis and neovascularization 71, 72. A clinical trial conducted by Huang *et al.* subcutaneously injected recombinant human GM-CSF to mobilize PB-MNCs. They found that intramuscularly-injected mobilized PB-MNCs improved ABI and blood perfusion in diabetic CLI patients 139. A phase I trial conducted by Murphy *et al*. showed that intramuscularly-injected autologous BM-MNCs increased ABI, blood perfusion, and amputation-free survival (AFS) while decreasing RP in patients with CLI 140. Two ongoing phase III clinical trials using intraarterially-injected autologous BM-MNCs cell suspension composed of several mature cell types (REX-001) are being held to investigate its efficacy and safety in treating diabetic CLI patients with Rutherford category 4 or 5 (NCT03111238 and NCT03174522, respectively). However, Lindemen *et al.* claimed that there is no clinical benefit to using BM-MNCs to treat non-reconstructable PAD patients 131.

In addition to MSCs and MNCs, other cells might also favor angiogenesis. For example, in a phase I clinical trial, a cell product composed of fully differentiated ECs overexpressing *ANG-1* and smooth muscle cells overexpressing *VEGF_165_* was administered intraarterially in “no-option” CLI patients. More than 70% of Rutherford 4 and 5 patients were free from amputation at a one-year time point. In addition, complete wound healing was observed in 50% of the patients with wounds. Despite its promising phase I clinical trial results, further clinical trials are required to confirm the benefits of this cell product 141. Another phase II clinical trial utilized intramuscularly-injected autologous blood-derived angiogenic cell precursor (ACP-01) to treat “no-option” CLI patients (NCT02551679); however, we could not find any information regarding the results at current. Meanwhile, a preclinical study conducted by Nakagami *et al.* transplanted autologous adipose-derived stem cells (ADSC) in a mouse HLI model and found that ADSC could favor angiogenesis in ischemic skeletal muscle tissue 142; while another preclinical study produced by Johnson *et al.* demonstrated that exosomes secreted by vascular progenitor cells could stimulate angiogenesis *in vivo* and* in vitro* 143. Furthermore, a preclinical study compared the angiogenic potential of exogenously administered ECs derived from embryonic stem cells (ESC-ECs) to ESCs, and results showed that ESC-ECs enhanced neovascularization and improved blood perfusion in the HLI mice model 144.

However, there are some obstacles to cell-based therapies that need to be overcome. First, MSCs poor retention and survival rates in the ischemic zone, and their low availability, especially in diseased states such as hyperglycemia, are major impediments to MSCs-based therapeutic angiogenesis 145, 146. Efforts have been made in preclinical settings to overcome these problems, for example, by combining MSCs-based treatment with gene-based therapy. Compared to untreated MSCs, the injection of engineered MSCs with a lentivirus to overexpress *VEGF-A* resulted in enhanced autocrine and paracrine function, further increasing blood perfusion in immunodeficient HLI mice 147. To solve the problem of MSCs poor survival rates in ischemic sites, Huang *et al.* tried to utilize biomaterial scaffolds mimicking ECM properties to improve MSCs adaptability. They used Nap-GFFYK-Thiol, a small molecule hydrogel, which could serve as a favorable niche for human placenta-derived MSCs to exert their paracrine function, and found that Nap-GFFYK-Thiol reduced MSCs apoptosis and maintained their undifferentiated state, resulting in significantly improved blood perfusion in HLI mice 146. Meanwhile, to improve MSCs availability, efforts such as hypoxia treatment, drug treatment, and the use of ECM scaffold have been made to optimize the expansion condition to increase MSCs population, migratory capabilities, and paracrine function in HLI model mice 148-150. Besides MSCs, ECs poor alignment in disturbed flow fields might lead to altered function and reduced survival. A preclinical study conducted by Huang *et al.* found that the alignment, function, and survival of ECs implanted in aligned nanofibrillar collagen scaffolds that mimic the structure of collagen bundles in blood vessels are improved in the ischemic sites of HLI mice 151.

Together, although preclinical studies and clinical trials claim that the efficacies of cell-based therapeutic angiogenesis are favorable; improvements in ABI and AFS only appear in selected patients 135. Besides the availability and survival rates of the cells used to induce therapeutic angiogenesis, different administration methods and patients' comorbidities, including genetic alterations, somatic mutations, and clonal hematopoiesis, are also major factors affecting clinical outcomes of cell-based therapeutic angiogenesis 131, 152, 153. Thus, promoting the number and quality of cells, optimizing the administration method, and analyzing individual risk factors are necessary prior to cell therapy.

### Protein-based therapy

Protein-based therapy is a straightforward method using recombinant proteins delivered locally or systemically to patients to induce angiogenesis through their direct functions in regulating and/or stimulating blood vessel formation 154. It does not involve gene transfer using virus or plasmid, or transplantation of cells into the host. Hence, compared to gene-based or cell-based therapy, protein-based therapy has been recognized as a safer and more effective method for therapeutic angiogenesis. Since the first clinical trial of a protein-based strategy using FGF-1 in 1998 155, numerous studies based on recombinant protein as well as structurally modified protein have been conducted to test their safety and efficacy in treating PAD; however, as shown in **Table 4**, only a few of them have entered clinical trial phases.

Protein therapy using FGF-2 is one of the most extensively studied protein-based therapeutic angiogenesis strategies, with promising outcomes such as high tolerance and increased blood flow seen in the phase I study of intraarterially-administered FGF-2 in patients with IC 100. The later phase II study indicated an improvement in peak walking time at 90 days, although this improvement did not last until 180 days 101. Meanwhile, Milton *et al.* conducted an early phase I trial on PAD patients using intravenously-administered angiotensin-(1-7), which has been known to protect against atherosclerosis in animal models by improving blood vessel function and reducing inflammation, on PAD patients (NCT03240068); however, the results of this study are not yet available. Another clinical trial was performed using GM-CSF, which contributes to the mobilization of EPCs; however, subcutaneously-administered GM-CSF did not significantly improve the walking performance of PAD patients 156.

Despite the benefits of protein-based therapy, as described above, in many cases, a large dose injection of protein is needed to effectively induce angiogenesis. This might induce immunogenicity proteinuria, an adverse event in which urine protein excretion exceeds 300 mg in 24 hours 157. Furthermore, their short *in vivo* half-lives, physical and chemical instability, as well as low bioavailability also contributed to inefficient outcomes in protein-based therapeutic angiogenesis 154, 158. To overcome these problems, researches based on computational design have been conducted to improve their delivery efficacy by developing sustained-release forms 159. Segers *et al.* added the sequences of self-assembling peptide nanofibers into recombinant SDF-1 to help the incorporation of this recombinant protein into nanofibers, thus avoiding rapid diffusion of SDF-1. This recombination strategy significantly improved blood flow and arteriogenesis in HLI mice 160.

In addition to the administration of angiogenic proteins, therapeutic angiogenesis induction using neutralization antibodies or proteins has also been reported. VEGF-A_165_b is an anti-angiogenic splicing form of VEGF-A_165,_ which suppresses canonical VEGF-A signaling by competitive interference with the VEGF receptor, and thus inhibits angiogenesis. Furthermore, VEGF-A_165_b is upregulated in PAD, leading to impaired VEGF-A signaling revascularization in HLI mice models 52. A preclinical study using HLI model mice showed a significant improvement in blood perfusion in the ischemic hindlimb intramuscularly administered with monoclonal antibody inhibiting VEGF-A_165_b 161, 162. Meanwhile, preclinical studies also showed that polarizing primary macrophages using lipopolysaccharide and interferon-gamma increased the expression level of the inflammatory cytokine interleukin-1β (IL-1β), thus inducing angiogenesis by activating VEGF-A transcription in the HLI mouse model.

Together, while preclinical trials demonstrate the positive effects of protein-based therapy in promoting angiogenesis, clinical trials still show some drawbacks of this approach. This includes adverse events, such as immunogenicity proteinuria, inefficient outcomes due to the short half-lives, poor physical and chemical properties of the proteins, and low bioavailability. Furthermore, the discovery of the anti-angiogenic VEGF-A splicing isoform, VEGF-A_165_b, complicates the understanding of PAD pathogenesis and treatment approach. Hence, more studies regarding the safety, sustained delivery, and mechanism of protein-based therapy to treat PAD are needed. Moreover, computational design to optimize the structure of the protein and increase their stability, such as conducted by Sun *et al.* in preclinical study using modified FGF2 for wound healing 163, also could help to solve the problem of the protein-based therapeutic angiogenesis.

### Small molecule drug-based therapy

Small molecule drugs offer more targeted signaling due to their small size and low molecular weight 164, 165. Furthermore, compared to proteins, the synthesis and purification of small molecule drugs are more accessible and cost-effective, as the molecule's structure is precisely known 166. There are three major sources of small molecule drugs: natural products, *de novo* synthesis of small molecule compounds or modification of previously existing small molecule compounds, and compounds that have been approved for treating other diseases. As will be discussed below, while numerous preclinical studies and clinical trials using small-molecule drugs for treating PAD have been conducted, the use of compounds that have been approved for treating other diseases might be a shortcut for developing an effective therapeutic strategy for treating PAD. Indeed, as shown in **Table 5**, most of the small molecule drugs that have emerged into clinical trials for treating PAD are those that have been approved for clinical use for treating other diseases.

Preclinical studies demonstrated that several active compounds extracted from traditional herbs and plants could promote angiogenesis. Zhang *et al.* found that baicalin, the major component found in the root of the traditional Chinese herb *Scutellaria baicalensis*, which is used to treat stroke, could promote VEGF expression in HUVECs and human fibroblast MRC-5 cell lines, suggesting the possibility of using baicalin to induce therapeutic angiogenesis in PAD 167. Meanwhile, Pignet *et al.* found that orally-administered resveratrol, a nutritional polyphenol that could provide beneficial effects on skin and could be found in more than 70 different plant species such as grapes, mulberries, and blueberries, promoted wound healing by reducing oxidative stress in mice models 168. Another promising active compound from the natural product is salidroside, which is an active compound derived from *Rhodiola rosea*, a plant commonly used as a traditional Chinese medicine to treat high-altitude sickness. Zhang *et al.* found that salidroside upregulated HIF-1α protein expression and then promoted VEGF-A, thus inhibiting hypoxia-induced cardiomyocytes necrosis and apoptosis 169. Zheng *et al.* found that salidroside treatment provided an anti-hypoxia effect by stimulating HIF-1α protein accumulation, but not HIF-2α protein, in human embryonic kidney fibroblast (HEK293T) and human hepatocellular carcinoma (HepG2) cell lines 170. Chen *et al.* found that intraperitoneally-administered salidroside promoted the expression of VEGF and increased skin flap angiogenesis in rat models 171. Studies by Zhang *et al.* revealed that targeting skeletal muscle cells with salidroside and its metabolite, tyrosol, significantly improved their proliferation and migration potentials, as well as their paracrine function. Furthermore, they demonstrated that intramuscular injection of salidroside and tyrosol into the gastrocnemius muscle of the ischemic hindlimb of HLI mice could enhance the formation of mature and functional blood vessels in non-diabetic and diabetic HLI mice models 75, 172, 173.

Another common strategy for searching novel small molecule drugs is by synthesizing and/or modifying small molecule compounds. Despite the beneficial effects of salidroside in inducing the formation of new blood vessels and in promoting blood perfusion recovery, as mentioned above, the concentration needed to exert these effects is high, hindering its potential clinical application. Recently, a preclinical study established by Liu *et al.* divided the functional groups of salidroside and synthesized more than 30 salidroside analogs by modifying its structure. The efficacy of these analogs in increasing HIF-1α transcriptional activity was analyzed, and salidroside analogs with significantly better stability, angiogenic property, and drug-likeness were determined based on a structure-activity relationship (SAR) study. The most optimized compound in this study could induce better neovascularization and blood perfusion recovery than salidroside in both non-diabetic and diabetic HLI mice at a significantly lower dose 56. Epigallocatechin-3-gallate (EGCG), the active compound extracted from green tea, has demonstrated antioxidative and anti-inflammatory functions. A preclinical study established by Duan *et al.* showed that by combining copper ions with EGCG into a metal-polyphenol capsule (Cu-EGCG), sustained released copper ions and EGCG from Cu-EGCG promoted blood perfusion recovery by scavenging ROS in HLI model mice 174. Another optimization of small molecule drugs for treating PAD was exerted by Hou *et al.* They synthesized small molecule compounds derived from carboxyethylpyrrole (CEP) protein adducts, which have been implicated in the induction of angiogenesis in pathological conditions, such as tissue ischemia 175. By screening the CEP analogs obtained, they found that intramuscular injection of CEP03, one of the analogs, could enhance EPCs migration and microvessel formation, thus subsequently promote angiogenesis and blood recovery rate of the HLI mice. This newly synthesized small molecule offers a more stable and easily absorbed option for the drug over CEP 166. L-arginine improves microcirculation by activating nitrogen monoxide production and stimulating capillary blood flow, thus it can probably improve the quality of life of patients with IC; however, its biosynthesizing rate *in vivo* is slow, leading to the urgent need for its supplementation in bodies. Moreover, orally supplemented L-arginine could easily be metabolized, further hindering its application in treating PAD. Optimization in drug delivery method and modification of the compound administered lead to the improvement of L-arginine-based therapeutic angiogenesis. To overcome the problem of oral administration of L-arginine, a phase II clinical trial using intravenously-delivered Unifuzol®, a drug-containing L-arginine, was conducted in 2019 in patients with IC to examine its effect on improving their walking distance (NCT03861416). Meanwhile, a clinical trial using orally administered L-citrulline, a natural precursor of L-arginine, which is metabolized to a lesser degree compared to L-arginine, has also been conducted (CIPER; NCT02521220).

Furthermore, a double-blind clinical trial showed that cilostazol, an anti-platelet drug with phosphodiesterase-3A (PDE-3A) inhibitory activity in platelets and vascular smooth muscle with anti-thrombotic and vasodilatory effects, could improve limb perfusion and peak walking time in patients with IC, most plausibly by upregulating EPC mobilization and increasing the expression of HGF, VEGF-A, and ANG-1 176, 177. Rivaroxaban is an inhibitor of clotting factor Xa that exerts anti-thrombotic activity by selectively blocking the active site of factor Xa 178. A phase III clinical trial showed that rivaroxaban could reduce the incidence of ischemic events in PAD patients, including acute limb ischemia 179, 180. Anti-cholesterol drugs, statins, which decrease cholesterol synthesis by competitively inhibiting endogenous cholesterol synthesis rate-limiting enzyme reductase, are also potential candidates for treating PAD 181. Statin administration could promote angiogenesis in HLI animal models by positively regulating eNOS-mediated vasodilation; furthermore, they could reduce the amputation rates in PAD patients 182-184. Sildenafil is a selective inhibitor of cyclic guanosine monophosphate (cGMP)-specific PDE type 5 (PDE5) and is an oral drug for treating erectile dysfunction 185. Low concentrations of PDE5 are also found in platelets, blood vessels, visceral smooth muscle, and skeletal muscle, suggesting that sildenafil might exert beneficial effects as anti-platelet, anti-thrombotic, and dilatation drugs 186, 187. A phase II clinical trial showed that orally administered sildenafil could efficiently improve symptoms and walking capacity in patients with stage 2 claudication. The clinical trial using this drug has now progressed into the stage of recruiting patients for the phase III trial (NCT03686306).

Preclinical studies have also revealed that small molecule drugs clinically approved for treating diabetes might also be effective in promoting therapeutic angiogenesis. Interestingly, these drugs induce therapeutic angiogenesis, not through their canonical anti-hyperglycemic mechanisms. Metformin, the first-line drug for type 2 diabetic mellitus, also has a specific regulatory effect on the process of angiogenesis. The research found that intraperitoneal injection of metformin exhibits potential for improving endothelial function and the wound-healing process in mice models without affecting their blood glucose levels 188. Recent studies have also revealed that another FDA-approved anti-diabetic class of drugs, gliflozins, which lower blood glucose by suppressing kidney glucose reabsorption by inhibiting sodium-glucose cotransporter 2 (SGLT2) 189, are also potential small molecule drug candidate for treating PAD. Despite the absence of SGLT2 in skeletal muscle cells, intramuscular injection of two members of gliflozin, namely dapagliflozin and sotagliflozin, into the ischemic site could induce the paracrine capacity of skeletal muscle cells, resulting in a significant increase in angiogenesis and blood perfusion recovery in diabetic HLI mice 74, 190. Similar to metformin, intramuscular administration of these drugs did not affect blood glucose levels, suggesting that their mechanism in inducing therapeutic angiogenesis is different from their mechanism for lowering blood glucose. Indeed, oral intake of dapagliflozin did not correlate with the risk of amputation, further supporting the distinct mechanism and effect induced by oral and intramuscular administration of this drug 191, 192. Furthermore, it is noteworthy that oral administration of another member of this class of drug, canagliflozin, increased the risk of amputation in patients with type 2 diabetes and impaired BM-MSCs function in HLI mice models 193-195, indicating that structural differences in small molecule drugs might cause different effects in treating PAD. Thus, further investigations regarding the SAR and the administration method of gliflozins for treating PAD need to be conducted.

Together, small molecule-based therapies could overcome drug delivery and the high-cost problems of other therapeutic angiogenesis strategies. Furthermore, the use of drugs clinically approved for other diseases might provide a shortcut for discovering potential therapeutic angiogenic agents. However, more clinical trials should be conducted to examine their efficacy. Moreover, as many of these drugs benefit PAD most likely through mechanisms distinct from their canonical, well-known ones, detailed studies regarding the molecular mechanisms underlying their effect in inducing therapeutic angiogenesis also need to be conducted. On the other hand, while structural optimization or combination with biomaterials and intensive preclinical and clinical studies are required, traditional herbs or plants provide numerous potential active compounds for treating PAD.

## Conclusion

Gene-, cell-, protein-, and small molecule drug-based therapeutic angiogenesis has emerged as an important potential strategy for treating PAD (**Figure 3**). Gene-based therapy uses genetic approaches to induce therapeutic angiogenesis. The first generation of gene-based therapeutic angiogenesis aims to induce the expression of single angiogenic factors, such as VEGF, HGF, and FGFs. The second generation, on the other hand, aims to induce therapeutic angiogenesis more efficiently by expressing two or more angiogenic factors simultaneously, or by expressing or suppressing upstream regulators of angiogenic factors, such as HIF-1α and the PHD family, as angiogenic is a complex process involving various vectors and cell types. Although some of the gene-based therapies have entered clinical trials, and two of them have been approved for clinical use, the concerns regarding their safety and delivery efficacy remain to be solved. Cell-based therapy has the potential to overcome the problem of the requirement of various angiogenic factors to induce efficient therapeutic angiogenesis in ischemic sites; however, it requires transplantation of viable stem or progenitor cells. These hurdles, along with the need for large amounts of cells and a lack of an appropriate niche in the transplantation sites, need to be overcome for further clinical use. Meanwhile, protein-based therapy, which introduces recombinant proteins to patients to induce angiogenesis, might benefit through their direct functions in regulating and/or stimulating blood vessel formation. However, this approach has several drawbacks, including adverse events, such as immunogenicity proteinuria, and properties of the proteins, such as short half-lives, poor physical and chemical properties, and their low bioavailability. Thus, further modifications and structure-activity relationship studies are needed to reduce their adverse effects and improve their drug-likeness. Meanwhile, due to its low-cost, small molecule drug-based therapy, which could alter the expression levels of angiogenic factors and thus improves angiogenesis in PAD patients, has attracted attention for its use in therapeutic angiogenesis. While many preclinical studies using small molecule compounds have been conducted, only a few of them have emerged into clinical trials at current, and one of the main problems to be solved is their chemical and physical characteristics as well as their drug-likeness.

To broaden the future application of therapeutic angiogenesis-based strategies in treating PAD, more sophisticated optimizations are needed to solve the problem of the gap between preclinical and clinical trial outcomes. First, delivery methods should be improved. This could be achieved by, for example, improving the specificity, stability, and biocompatibility of the gene or protein being transferred by modifying their structures or combining them with biomaterials; optimizing the gene or protein being transferred by modifying their structures or combining them with biomaterials; enhancing the viability of the cells used in cell-based therapy; and developing a sustained-release delivery system using appropriate biomaterials. Second, the availability of therapeutic angiogenesis agents should be increased to reduce costs, for example, by optimizing the extraction and purification methods of vectors and proteins or by increasing the availability of cells using conditions mimicking *in vivo* circumstances. In terms of availability and cost performance, small molecule drugs might offer a more economical strategy, as their synthesis and purification are easier and more cost-effective. Third, as the innate angiogenesis potential, which determines the recovery ability, differs among species and even among patients, more appropriate PAD animal models that could reflect different recovery abilities and stages of disease progression should be established. Furthermore, more appropriate criteria should also be used as standards for selecting patients appropriate for different therapeutic angiogenesis approaches.

Drugs clinically approved for other diseases might offer a shortcut for clinical translation of therapeutic angiogenic strategy. Although they should also undergo a complete clinical trial to re-examine their safety and efficacy in treating PAD, especially when different administration methods and/or doses are used, it will usually take a significantly shorter time than unapproved compounds. However, the molecular mechanisms underlying the therapeutic angiogenesis effects of these drugs need to be elucidated, as many of them exert beneficial effects on PAD patients, not through their known canonical pathways. Attention should also be paid to delivery methods and drugs with similar structures, as slight differences in structure and/or delivery method might result in totally different therapeutic effects. Furthermore, the combination of small molecule drugs and biomaterials might benefit future clinical applications of small molecule drug-based therapy in PAD patients.

Overall, while further preclinical investigations and more clinical trials are needed to develop safe, effective, and economic-friendly strategies, current preclinical and clinical trials have already demonstrated the promising effects of therapeutic angiogenesis, thus highlighting its importance as a potential strategy for treating PAD.

## Figures and Tables

**Table 1:** Clinical trials using gene-based therapy

Administered drug	Phase	Administration	Target patient	Reference
Adenoviral vector encoding *VEGF-A_121_*	I	Intramuscular	PAD patients with IC or RP	106
Plasmid encoding *VEGF-A_165_* (Neovasculgen)	III	Intramuscular	Limb ischemia of atherosclerotic genesis	28
Plasmid encoding human *HGF*	I/II	Intramuscular	CLI	98, 99
Plasmid encoding human *HGF* (pUDK-HGF)	II	Intramuscular	CLI	97
Plasmid encoding human *HGF* (pUDK-HGF)	III	Intramuscular	CLI	CTR20181274
Plasmid encoding human *HGF_728_* and *HGF_723_* (VM202)	II	Intramuscular	PAD	NCT03363165
Plasmid encoding *HGF_728_* and *HGF_723_* (NL003)	III	Intramuscular	CLI	NCT04274049
Plasmid encoding human *FGF* (NV1FGF)	II	Intramuscular	CLI	113
Recombinant Sendai virus encoding human *FGF-2* (SeV-hFGF2/dF)	I	Intramuscular	Peripheral arterial occlusive disease	NCT03668353
Adeno-associated virus encoding human hTERT (AAV-hTERT)	I	Intravenous	CLI	NCT04110964
Plasmids encoding human *VEGF-A_165_* and *HGF*	I	Intramuscular	CLI patients with diabetes	119
Adenoviral vector encoding human *HIF-1α*	I	Intramuscular	Advanced atherosclerosis and tissue ischemia	89, 119
Adenoviral vector encoding human *HIF-1α*	II	Intramuscular	PAD patients with IC	131

Abbreviation: CLI: critical limb ischemia; IC: intermittent claudication; PAD: peripheral artery disease; RP: rest pain.

**Table 2:** Clinical trials using cell-based therapy

Administered drug	Phase	Administration	Target patient	Reference
Allogeneic BM-MSC	II	Intramuscular	CLI (Rutherford 4-5)	137
Allogeneic placental-derived mesenchymal-like cells	III	Intramuscular	CLI due to atherosclerosis (Rutherford 5)	138
GM-CSF-mobilized PB-MNC	-	Intramuscular	CLI patients with diabetes	139
Autologous BM-MNC	III	Intramuscular	CLI (Rutherford 4-5)	140
Autologous BM-MNC (REX-001)	III	Intraarterial	CLI patients with diabetes (Rutherford 4)	NCT03111238
Autologous BM-MNC (RvEX-001)	III	Intraarterial	CLI patients with diabetes (Rutherford 5)	NCT03174522
BM-MNC	III	Intramuscular	Non-reconstructable PAD	131
ECs expressing *ANG-1*, combined with autologous smooth muscle cells expressing *VEGF-A_165_*	I	Intraarterial	CLI	141
Autologous blood-derived angiogenic cell precursor (ACP-01)	II	Intramuscular	CLI (except advanced CLI)	NCT02551679

Abbreviations: BM-MNC: bone marrow-derived mononuclear cells; BM-MSC: bone marrow-derived mesenchymal stem cells; CLI: critical limb ischemia; ECs: endothelial cells; GM-CSF: granulocyte-macrophage colony-stimulating factor; PAD: peripheral artery disease; PB-MNC: peripheral blood-derived mononuclear cell.

**Table 3:** Advantages and disadvantages of allogeneic and autologous cell-based therapy

	Allogeneic	Autologous
Safety	Possible immunoreactivity and incompatibility 132	Immunocompatible as the cells are obtained from the host itself 132
	Invasive cell isolation process to donors; in need of healthy and eligible donors 137	Additional invasive cell isolation process to the patient 131
Efficacy	Robust cell availability and potency as cells are collected from healthy donors 132	Possibility of inconsistent cell availability and potency, depends on the host's condition 133

**Table 4:** Clinical trials using protein-based therapy

Administered drug	Phase	Administration	Target patient	Reference
FGF-2 (bFGF)	I	Intraarterial	Atherosclerotic PAD with IC	100
Recombinant FGF-2	II	Intraarterial	IC	101
Angiotensin 1-7	I	Intravenous	PAD	NCT03240068
GM-CSF	N/A	Subcutaneous	PAD	156

Abbreviations: GM-CSF: granulocyte-macrophage colony-stimulating factor; N/A: not applicable; IC: intermittent claudication; PAD: peripheral artery disease.

**Table 5:** Clinical trials using small molecule drug-based therapy

Administered drug	Phase	Administration	Target patient	Reference
L-arginine (Unifuzol®)	II	Intravenous	PAD	NCT03861416
L-citrulline	N/A	Oral	IC	NCT02521220
Cilostazol	-	Oral	IC	176
Rivaroxaban	III	Oral	PAD	179, 180
Statin	-	Oral	PAD	184
Sildenafil	III	Oral	IC	NCT03686306

Abbreviations: IC: intermittent claudication; PAD: peripheral artery disease; N/A: not applicable.

## Abbreviations

ABI: ankle-brachial index
ADSC: adipose-derived stem cells
AFS: amputation-free survival
AGEs: advanced glycation end-product
AGGF1: angiogenic factor with G-patch and Forkhead-associated domain 1
ANG-1: angiopoietin-1
BM-MNC: bone marrow-derived mononuclear cell
BM-MSCs: bone marrow-derived mesenchymal stem cells
CEP: carboxyethylpyrrole
cGMP: cyclic guanosine monophosphate
CLI: critical limb ischemia
ECs: endothelial cells
ECM: extracellular matrix
EGCG: epigallocatechin-3-gallate
eNOS: endothelial nitric oxide synthase
EPCs: endothelial progenitor cells
ESC: embryonic stem cell
FGF-2: fibroblast growth factor-2
GM-CSF: granulocyte-macrophage colony-stimulating factor
HEK293T: human embryonic kidney fibroblast
HepG2: human hepatocellular carcinoma
HGF: hepatocyte growth factor
HIF-1: hypoxia-inducible factor-1
HLI: hindlimb ischemia
HO-1: heme oxygenase-1
hTERT: human telomerase reverse transcriptase
IC: intermittent claudication
IL-1β: interleukin-1β
MALE: major adverse limb events
MCP-1: monocyte chemoattractant protein 1
miRNA: micro RNA
MMP: matrix metalloproteinase
PAD: peripheral artery disease
PB-MNC: peripheral blood-derived mononuclear cell
PDE: phosphodiesterase
PDE5: PDE type 5
PDGF-BB: platelet-derived growth factor BB
PHD: prolyl-hydroxylase domain
ROS: reactive oxygen species
RP: rest pain
SAR: structure-activity relationship
SDF-1: stromal cell-derived factor-1
SGLT2: sodium-glucose cotransporter 2
VEGF: vascular endothelial growth factor
VSMCs: vascular smooth muscle cells
